# A practical example: how alcohol screening and brief intervention can work in a real-life New Zealand primary-care environment

**DOI:** 10.1186/1940-0640-7-S1-A52

**Published:** 2012-10-09

**Authors:** Susan Paton, John McMenamin, Kristen Maynard

**Affiliations:** 1 Alcohol Advisory Council of New Zealand, Wellington, New Zealand; 2 Whanganui Regional Primary Health Organization, Whanganui, New Zealand

## 

New Zealand has a high level of acute alcohol-related harm compared with other countries. In the most recent alcohol use survey, 61.6% of New Zealand drinkers aged 16-64 years reported heavy episodic drinking at least once in a 12-month period, and 12.6% reported weekly heavy episodic drinking. There is substantial multinational evidence, and growing New Zealand evidence, showing that alcohol screening and brief intervention (SBI) in primary-care and emergency-department (ED) settings is an efficacious and cost-effective approach to reducing alcohol-related harm among people with heavy episodic drinking. Despite this, SBI is significantly underutilized in New Zealand. This implementation description demonstrates how SBI has been integrated successfully into general practice in the Whanganui region of New Zealand. In 2010, the Alcohol Advisory Council of New Zealand (ALAC) provided support to the Whanganui Regional Primary Health Organization (WRPHO) to implement an alcohol SBI approach that involved asking all patients ≥15 years about their alcohol consumption, offering brief advice to those identified as risky drinkers, and, where appropriate, referring patients to specialist services. This approach used a reminder system on the front page of electronic patient notes and an advanced form in the patient management system. In just under a year, 43% of all patients ≥15 years enrolled in the WRPHO had been asked about their alcohol consumption, with 36 percent of these patients receiving a brief intervention or specialist referral. The WRPHO project highlights how SBI can work in a New Zealand primary-care setting and its potential efficacy for reducing alcohol harm.

